# How Many Fenestrations Should I Make When Placing a Baerveldt Glaucoma Implant? A Laboratory Study

**DOI:** 10.18502/jovr.v18i2.13181

**Published:** 2023-04-19

**Authors:** Michael C Yang, Christopher D Yang, Ken Y Lin

**Affiliations:** ^1^Gavin Herbert Eye Institute, Department of Ophthalmology, University of California, Irvine, California; ^2^Department of Biomedical Engineering, University of California, Irvine, California; ^4^https://orcid.org/0000-0003-3292-8578; ^5^https://orcid.org/0000-0002-6467-7219

**Keywords:** Baerveldt, Drainage Implants, Fenestration, Glaucoma, Opening Pressure

## Abstract

**Purpose:**

This study investigates the effect of one versus two fenestrations on both fluid egress and opening pressure from a non-valved glaucoma implant.

**Methods:**

In this laboratory study, we used an *in vitro* closed system comprised of ligated silicone tubing connected to a fluid reservoir and manometer to simulate the tubing found in a Baerveldt glaucoma drainage implant. Fenestrations were created using an 8-0 Vicryl TG140-8 suture needle. Main outcome measures included volume of fluid egress and fenestration opening pressures, which were measured via micropipette and increasing pressure until fluid egress was observed.

**Results:**

No significant difference was observed in fluid egress between tubing with one versus two fenestrations at pressures ≤ 40 mmHg. At 50 mmHg, a statistically significant difference was observed in fluid egress between tubing with one versus two fenestrations (*P* < 0.05). The first fenestration opened at 10.5 ± 3.77 mmHg and the second fenestration opened at 28.83 ± 5.09 mmHg (average ± standard deviation).

**Conclusion:**

Our *in vitro* findings suggest there may exist a critical pressure > 40 mmHg at which the second fenestration starts to play a significant role in fluid drainage. There may be no difference in the amount of fluid egress and effect on intraocular pressure between one or two tube fenestrations when preoperative intraocular pressure is ≤ 40 mmHg.

##  INTRODUCTION

In the recent years, glaucoma drainage implants (GDIs) have become a mainstay in surgical glaucoma management becoming the preferred option over trabeculectomy in a growing number of glaucoma practices.^[1]^ GDIs share a common anatomy comprising of a silicone tube connected to an endplate. The silicone tube is surgically inserted into the eye to allow access to the aqueous humor, and the endplate is fixed to the sclera and covered with conjunctiva and Tenon's capsule. Like trabeculectomy, GDIs are considered penetrative glaucoma surgeries that create a *de novo* pathway for aqueous drainage.

The two most common GDIs on the market are the Ahmed glaucoma drainage implant (AGI; New World Medical, Rancho Cucamonga, California) and the Baerveldt glaucoma drainage implant (BGI; Johnson & Johnson, Santa Ana, California). When compared to the AGI, the BGI results in a significantly lower mean intraocular pressure (IOP) with lower rates of failure. However, the BGI carries a higher risk of postoperative hypotony, in part because it does not have a valve mechanism.^[2,3]^ Valved implants generally have a pressure floor below which aqueous flow through the implants is disabled. The Krupin implant utilizes a unidirectional valve that opens when IOP is > 11 mmHg.^[4]^ The valve mechanism of the AGI involves thin silicone membranes that open, via the Venturi effect, when IOP is > 8–12 mmHg.^[5]^ On the other hand, non-valved implants, like the BGI, allow for unrestricted flow of aqueous humor and rely on encapsulation around the endplate for flow resistance.^[6]^ For this reason, flow through non-valved glaucoma implants must be restricted in the immediate postoperative period before encapsulation has occurred. One common restriction method is to ligate the silicone tube with dissolvable suture. It is common practice to fenestrate the ligated tubes to allow for some degree of aqueous humor drainage while the capsule is maturing.^[6]^

Little consensus exists regarding the number and manner with which fenestrations are created in non-valved GDIs. Similarly, no heuristics exist to guide how fenestrations should or should not be modified based on preoperative IOP. Such heuristics, even if theoretical or based on *in vitro* observations, are nevertheless important because they may help guide clinicians to achieve a more stable and predictable IOP during the immediate postoperative period. Moreover, only a few published experiments evaluating the number of fenestrations and their effects on outflow facility and IOP in *in vitro* and *ex vivo *systems have been performed, with equivocal results.^[7,8]^ In an *in vitro* study of a ligated BGI with four fenestrations using a 7-0 Vicryl TG140-8 needle, the volume of fluid egress was found to positively correlate with simulated IOP.^[8]^ In an *ex vivo* study of porcine eyes, three fenestrations with a 7-0 Vicryl TG140-8 needle led to a significantly lower final IOP compared to a single fenestration after 15 min with an initial IOP of 50 mmHg.^[7]^ Additional studies are needed to evaluate the wide spectrum of fenestration possibilities and their effects on fluid egress.

In our study, we utilize an *in vitro* apparatus as a model for ligated BGI tubing to evaluate fluid efflux with one versus two fenestrations created with a Vicryl TG140-8 needle at discrete intra-tubular pressures. In addition to quantifying the volume of fluid egress, we also identify an opening pressure at which fluid outflow begins from each fenestration. We hypothesized that the number of fenestrations in the tubing does not significantly affect the volume of fluid efflux until a critical intra-tubular pressure threshold is reached.

##  METHODS

### Glaucoma Drainage Implant Experimental Apparatus

**Figure 1:**
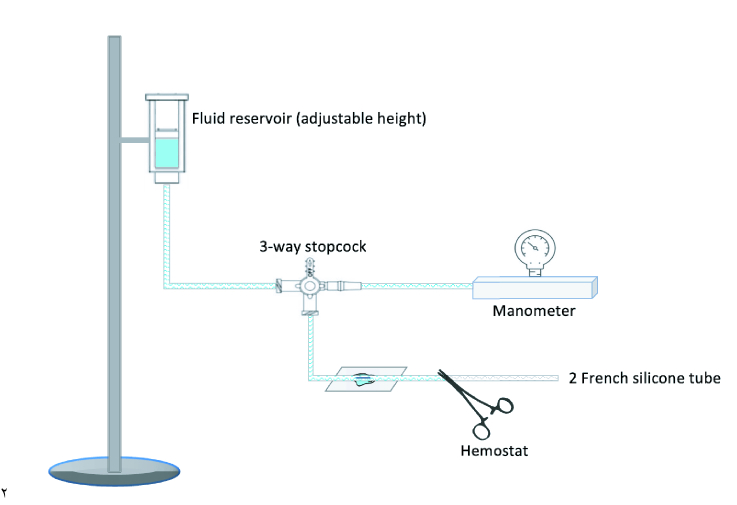
Diagram of *in vitro* experimental setup modeling a ligated silicone tube of a non-valved glaucoma drainage implant.Two French silicone tubing connected to a 27-gauge cannula attached to a three-way stopcock. The open end of the silicone tube was clamped with a hemostat. The other two ends of the three-way stopcock were attached to intravenous tubing with a fluid reservoir and to a manometer.

An *in vitro* experimental apparatus was created as a model for ligated BGI tubing [Figure 1]. Non-sterile silicone tubing (Access Technologies, 2 French silicone catheter, Model BC-2S), with an internal diameter of 0.3 mm and outer diameter of 0.6 mm, was used to simulate the silicone tubing attached to the Baerveldt glaucoma drainage implant (0.30 × 0.63 mm). The silicone tubing was connected to the system by a 27G cannula (Eagle Labs, 27ga × 1” cannula, Model 113-27NS) attached to a three-way stopcock (Medex, 3-Way Stopcock, Model MX4311L). The open end of the silicone tube was clamped with a hemostat to create a closed system. The other two ends of the three-way stopcock were attached to intravenous tubing (Baxter, Continu-Flo Solution Set, Model 2C8537s) with a 50 mL syringe as a fluid reservoir (BD, 50 mL syringe Luer-Lok Tip, Model 309653) and to a manometer (Omega, absolute pressure meter, Model HHC281). The fluid reservoir was filled with a balanced salt solution (Alcon, BSS Sterile Irrigating Solution, Model 9012632-1115).

### Tube Fenestration and Fluid Egress Measurement at Variable IOP 

**Figure 2:**
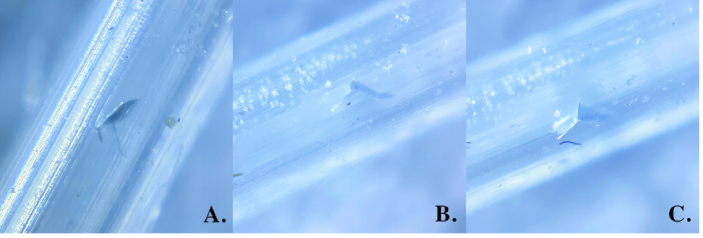
Microscopic images of fenestrations in silicone tubing*.* Fenestrations were created with 7-0 Vicryl on a TG140-8 spatulated needle. (**A)** Uniplanar fenestration structure. (**B)** Biplanar fenestration structure. (**C)** Triplanar fenestration structure.

A simulated IOP (or intra-tubular pressure) was held constant at a predetermined level by adjusting the height of the fluid reservoir (20, 30, 40, and 50 mmHg). Tube fenestrations were created using a spatulated suture needle (Ethicon, 7-0 Vicryl, TG140-8 needle) by entering perpendicularly to the center of and passing through the front and far side of the tubing. The two fenestrations were approximately 1 mm apart. Two horizontal slit openings (parallel to the walls of the tube, front and far sides of the tube) were created with a single fenestration [Figure 2]. Two fenestrations resulted in four horizontal slit openings. Care was taken to enter and exit along the same needle path to avoid enlarging the fenestration. The total volume of egressed fluid was measured from both fenestrations after 5 min using Beta-Pette micropipettes to the nearest microliter. Four trial measurements were obtained for each experimental replicate; each trial was conducted with new silicone tubing and newly created fenestrations [Table 1]. A total of 32 trial measurements were obtained.

### Opening Pressure Measurement

Simulated IOP (or intra-tubular pressure) was initially held at atmospheric pressure. Two fenestrations were then created in the manner as described in “Measuring the Volume of Fluid Egress at Variable IOP.” Subsequently, the hydrostatic pressure was gradually increased by raising the reservoir height at an approximate rate of 1 mmHg per sec. The intra-tubular pressure was increased until both fenestrations were open. The opening pressure of the fenestration was defined as the pressure required to induce approximately 5 µl of fluid efflux, given that this volume of fluid would be visible underneath the microscope. Five trial measurements were obtained for each of the six experimental replicates; each replicate was conducted with new silicone tubing and newly created fenestrations.

### Statistical Analysis

Data were recorded in an Excel spreadsheet and statistical analysis was performed with an unpaired Student's *t*-test like other studies comparing similar outcomes.^[7]^ *P*-values < 0.05 were considered statistically significant. Imaging of the fenestrations were obtained with a digital camera (iPhone 12 Pro, Apple, Cupertino, United States) equipped with a microscope attachment (DIPLE Lux, SmartMicroOptics, Genoa, Italy).

##  RESULTS

In the 20-mmHg trial, a single fenestration resulted in a mean fluid egress of 113.5 µL, two fenestrations 159 µL; not significantly different with a *P*-value of 0.16 [Table 1]. In the 30-mmHg trial, a single fenestration resulted in a mean fluid egress of 188.25 µL, two fenestrations 263.5 µL; not significantly different with a *P*-value of 0.15. In the 40-mmHg trial, a single fenestration resulted in a mean fluid egress of 213.75 µL, two fenestrations 293.75 µL; not significantly different with a *P*-value of 0.08, but notably trending toward significance. In the 50-mmHg trial, a single fenestration resulted in a mean fluid egress of 247.75 µL, two fenestrations 548.75 µL; notably different with a significant *P*-value of 0.02.

**Table 1:** Fluid egress from silicone tube with one versus two fenestrations at varying simulated intraocular pressures after 5 min. Fenestrations were created with 7-0 Vicryl on a TG140-8 spatulated needle.


**Simulated intraocular pressure (mmHg)**	**Number of fenestrations**	**Mean fluid egress at 5 min (µL) ± standard deviation**	**Minimum fluid egress at 5 min (µL)**	**Maximum fluid egress at 5 min (µL)**	***P*** **-value**
20 mmHg	1	113.5 ± 23.3	82	130	0.156
	2	159 ± 83.5	87	235	
30 mmHg	1	188.25 ± 128.85	53	350	0.149
	2	263.5 ± 10.01	235	283	
40 mmHg	1	213.75 ± 87.18	135	295	0.083
	2	293.75 ± 75.66	235	380	
50 mmHg	1	247.75 ± 124.89	144	428	0.021
	2	548.75 ± 174.81	505	620	
	
	
*mmHg, millimeters of mercury; µL, microliters					

**Figure 3:**
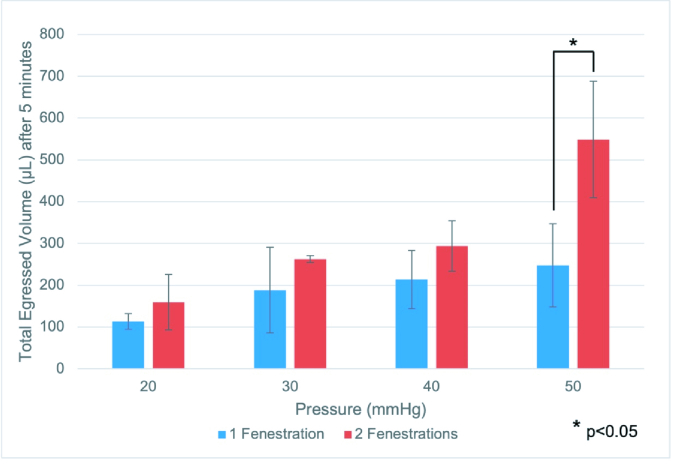
Fluid egress from silicone tube with one versus two fenestrations at varying simulated intraocular pressures after 5 min. Blue bar indicates one fenestration. Red bar indicates two fenestrations. Fenestrations were created with 7-0 Vicryl on a TG140-8 spatulated needle. *mmHg, millimeters of mercury; µL, microliters

Although mean fluid egress trended toward increasing with an additional fenestration, it was only statistically significant in the 50-mmHg group [Figure 3].

The first fenestration opened at 10.5 mmHg (range: 6–21 mmHg), while the second fenestration opened at 28.83 mmHg (range: 23–41 mmHg) with a *P*-value of < 0.001 [Table 2]. Notably, the first fenestration to open was not always the one closest to the fluid source.

**Table 2:** Opening pressure of first and second fenestration of silicone tube.Fenestrations were created with 7-0 Vicryl on a TG140-8 spatulated needle.


	**Mean opening pressure ± standard deviation (mmHg)**	**Minimum opening pressure (mmHg)**	**Maximum opening pressure (mmHg)**	***P*** **-value**
Fenestration #1	10.5 ± 3.77	6	21	< 0.001 (1.76E-19)
Fenestration #2	28.83 ± 5.09	23	41	
	
	
*mmHg, millimeters of mercury; E, 10 ${}^{x}$				

##  DISCUSSION

The salient findings of our study are as follows: (1) at a simulated intraocular pressure of 50 mmHg, there is a statistically significant difference in total volume of egressed fluid from the silicone tube with one versus two fenestration(s); (2) there is no significant difference if simulated IOP is 40 mmHg or less (the 40 mmHg group was very close to being statistically significant with a *P*-value of 0.08); (3) in the opening pressure trials, the second fenestration opened at a pressure of 28.83 ± 5.09 mmHg. The last two observations suggest that a critical opening pressure exists between 40 and 50 mmHg beyond which the second fenestration significantly contributes to fluid drainage out of the silicone tube. It is possible that the critical pressure may actually exist at a pressure between 30 and 40 mmHg, given that the second fenestration opens at approximately 29 mmHg. These findings suggest that the creation of a second fenestration in the ligated Baerveldt tube does not result in a significant decrease in IOP if the preoperative IOP is < 40 mmHg. In other words, single fenestration should be sufficient when the preoperative IOP is < 40 mmHg. Our *in vitro* experiment may provide some guidance to glaucoma surgeons when deciding the number of fenestrations to create intraoperatively. Yet, given the inherent limitations of an *in vitro* study, the results reported here should be considered in the broader context of surgeons' clinical and surgical expertise.

Olayanju et al characterized the outflow facility of a tube system with constant intraocular pressure and fenestrated with varying needles and blades.^[8]^ They found that the outflow facility (mL/min/mmHg) was mainly dependent on intraocular pressure and did not significantly change by external weight on the tube (e.g., scleral patch graft). However, they made the observation that the microarchitecture of the fenestrations was widely variable; the same external forces (e.g., scleral patch graft) could either reinforce fenestration closure or hold the fenestrations open. The fenestrations created by the 7-0 Vicryl TG140-8 needle appeared to exhibit the lowest outflow facility when compared to openings created by a 15 blade and 9-0 nylon CS140-6 needle with a suture stent. However, the authors did not investigate differences in volume outflow between varying numbers of Vicryl needle fenestrations.

Honda et al utilized an *ex vivo *experimental set up with pig eyes and a syringe pump perfusing fluid into the system at the same rate as aqueous production (200 µL/hr).^[7]^ Various needles were used to create one or three fenestrations (7-0 Vicryl, 7-0 PDS, 5-0 PDS, 3-0 PDS). After fenestrations were created, IOP was compared between needle types after 15 min of perfusion with a starting IOP of 50 mmHg. Only the 7-0 Vicryl group had a significantly lower final IOP between the one and three fenestration subsets. However, when comparing the IOP curves between tubes with one versus three fenestrations, the slope of IOP decline in the tube with three fenestrations was steeper between approximately 35–50 mmHg. Interestingly, when IOP was < 35 mmHg, both tubes (one and three fenestrations) appeared to exhibit similar slopes of IOP decline. This suggests that Honda et al also identified a critical opening pressure (approximately > 35 mmHg) at which the second and third fenestrations begin to significantly contribute to fluid drainage out of the tube. This critical opening pressure phenomenon may be explained by the elasticity of the silicone tubing. In the absence or insufficiency of an internal fluid load, hydrodynamic pressure cannot overcome the elasticity of the silicone, and the tube will self-seal.

Like other reports evaluating glaucoma tube shunt fenestrations, our results varied widely between each trial.^[7,8]^ This may be due to microscopic variations in surgical technique resulting in a variety of differences in microarchitecture between fenestrations [Figure 2]. We found that fenestrations with the 7-0 Vicryl TG140-8 needle could take the form of a uniplanar, biplanar, or triplanar structure. Certainly, other microarchitecture configurations are possible. It would be valuable to characterize the microstructure of each fenestration (e.g., uniplanar, biplanar, triplanar, etc.) and evaluate the differences in opening pressure and fluid egress of each microstructure type. Although our results do not consistently demonstrate this, other authors have found that multiple fenestrations lead to a wider standard deviation of results compared to a single fenestration.^[7]^ Presumably, with an increased number of fenestrations, microarchitecture variability also increases. For example, the distance between each fenestration and the location of the fenestrations in relation to the scleral patch graft may affect opening pressure and/or fluid egress. Honda et al also found that round needles (e.g., PDS needles) allowed for more consistent fenestration construction and more predictable experimental outcomes,^[7]^ further emphasizing the importance of fenestration microarchitecture.

The limitations of our study were that we utilized an *in vitro* experimental apparatus instead of pig or human eyes. Our model recapitulated a real eye but assumed no outflow via the traditional or uveoscleral pathways, and no peritubular flow. As with any *in vitro* model, our system does not perfectly mimic the normal physiology of aqueous drainage. To avoid variability in our measurements, we elected to hold the pressure at a constant level; however, in a human eye, IOP would decrease as aqueous exits. In other words, the physiologic flow rate would be dynamic and decelerate as the pressure falls below the opening pressures of the fenestrations. Additionally, we utilized balanced salt solution in place of aqueous humor. Prior studies have indicated that aqueous humor, with its various proteins and blood products, can occlude fenestrations.^[9]^ Aqueous humor also has a different viscosity from balanced salt solution and, as such, the actual opening pressure of an implanted BGI may be slightly different; however, we posit that the relatively small contribution from the second fenestration in most of the IOP ranges tested still holds clinically. It is important to keep in mind that the critical pressures reported in this study are likely lower than what would be seen in patients; the effect of episcleral fibrosis was not accounted for in this study. Moreover, our model does not include a simulated scleral patch graft. In theory, the scleral patch graft may apply external pressure to the tubing, either facilitating flow or blocking the fenestrations.^[8]^ However, prior studies (Olayanju et al) simulated the presence of the patch graft with external weights and found no significant difference in outflow facility of the tubing.

The apparent difference between our observed critical pressure ( > 40 mmHg) and the opening pressure of the second fenestration (approximately 30 mmHg) may be explained by the design of our fluid egress experiments, in which simulated IOP was increased in 10 mmHg intervals. These intervals do not precisely capture the pressure at which egress saturates. Future experiments can be performed to expand on our study by conducting experiments between the 10 mmHg intervals, determining the effect of interfenestration distance on opening pressure, studying the effect of fenestration numbers greater than two, and quantifying the microarchitecture of manually created fenestrations.

Our study demonstrates no significant difference in fluid outflow from two French silicone tubing between one or two fenestrations created by a 7-0 Vicryl TG140-8 needle at simulated IOPs ≤ 40 mmHg in an *in vitro* setting. The second fenestration has an opening pressure of approximately 29 mmHg but may not induce a significant effect on outflow until IOP is > 40 mmHg. Our study is limited by several aforementioned factors which may restrict its translation to clinical practice in patients. However, assuming the egressed volume of fluid correlates with IOP, our findings suggest that the creation of more than one fenestration in BGI tubing may not have a significant effect on postoperative IOP unless preoperative IOP is > 40 mmHg.

##  Financial Support and Sponsorship

KYL is a recipient of the Clinician Scientist Award from the American Glaucoma Society. The Gavin Herbert Eye Institute is a recipient of an unrestricted grant from Research to Prevent Blindness (RPB).

##  Conflicts of Interest

No conflicts exist for any author.
